# Minimally-invasive tubular resection of thoracolumbar intradural schwannoma

**DOI:** 10.3171/2023.6.FOCVID2335

**Published:** 2023-10-01

**Authors:** Maya Harary, Diana Chang, Irene Say, Daniel C. Lu

**Affiliations:** 1 Department of Neurosurgery, University of California, Los Angeles, California; and; 2 Department of Neurosurgery, University of California, San Francisco, California

**Keywords:** minimally invasive, tubular, intradural extramedullary, IDEM, schwannoma

## Abstract

Minimally invasive surgical (MIS) approaches to the spine are increasingly adopted for intradural pathology. In this setting, they may especially be useful to minimize risk of CSF leakage due to the decreased disruption to paraspinal musculature and minimal dead space. Herein, the authors demonstrate their technique for the resection of an intradural thoracolumbar schwannoma in a 30-year-old woman via an MIS approach using a nonexpandable tubular retractor. Salient points include the use of bayonetted instruments and the technique for dural closure in a small corridor. Indications for this technique are discussed in the context of a series of patients with intradural extramedullary lesions.

## Transcript

This video will demonstrate a minimally invasive tubular resection of a thoracolumbar intradural schwannoma. MIS approaches to the spine are increasingly popular. These are associated with expedited recovery, decreased narcotic usage, decreased complications, superior clinical outcomes, improved cosmesis, and patient satisfaction.^1–3^

For intradural pathology, these techniques may be especially useful to minimize risk of CSF leak due to the decreased disruption to paraspinal musculature and minimal dead space. While several have described the use of mini-open or expandable retractor approaches,^3–5^ purely nonexpandable tubular approach to intradural lesions are less common.^1,6^ Here we present our technique for tubular MIS resection of an intradural schwannoma.

### 1:01 Case Presentation.

The patient is 30-year-old female with history of type 1 diabetes who presented with 3 months of lower-back pain radiating to the left leg, as well as intermittent episodes of lower-body numbness triggered by sneezing.

On neurological exam, she was full strength throughout, but did have decreased sensation in left leg in a nondermatomal distribution. Imaging showed a contrast-enhancing, intradural extramedullary lesion at the thoracolumbar junction, eccentric to the left. Differential diagnosis at the time was a schwannoma versus myxopapillary ependymoma.

### 1:32 Operative Setup and Exposure.

The patient was positioned prone on a Jackson table, neuromonitoring was used, and the operative level was identified using fluoroscopy. An incision was made 1.5 cm left of midline. The dissection was continued subfascially. Serial tubular dissection (Medtronic METRx) was carried out over the T12 lamina to final 22-mm tube diameter and 60-mm length. The tubular system was secured in place, the microscope was brought into the field, and a full T12–L1 laminectomy was performed.

### 1:59 Dural Opening.

At the end of initial exposure, we see the dural sac, with screen left representing the cephalad direction.

A midline dural incision is made using a 15 blade, and subsequently extended cephalad and caudad by cutting over a right-angle hone or blunt probe. Care is taken to preserve the arachnoid. The dura is tacked up bilaterally.

### 2:39 Intradural Dissection and Tumor Resection.

At this point, we transition to bayonetted instruments when available to aid visualization. The arachnoid is sharply dissected. Gentle retraction of the nerve roots reveals the soft tumor mass ventrolaterally.

Circumferential dissection using blunt dissectors is done to identify the rostral and caudal poles of the tumor. A patty is placed on either end. Neural elements are seen posterolaterally to the tumor on either side, and the ventral dural is identified behind it.

After the extents of the tumor were identified, it is gently elevated out of the dural opening. This helps identify the afferent and efferent nerve rootlet. Care is taken to ensure the rootlet is indeed involved with the tumor and is not en passage. The rostral and caudal rootlets are stimulated to confirm no associated motor function. These are then coagulated and cut, and the free tumor mass is removed.

### 5:42 Closure.

After hemostasis is achieved, attention is turned to closure. The dura is reapproximated and closed with AnastoClips GC (LeMaitre). Prior to the final closure, the dural sac is filled with saline. The final clips are then applied, and a Valsalva maneuver is done to confirm dural closure. The dura is then covered with DuraSeal Exact (Integra). The tubular retractor is removed under the microscope to ensure hemostasis of the retractor tract. The soft tissue and skin are then closed in the usual fashion.

### 6:26 Outcome.

The patient was kept on bedrest overnight, was mobilized liberally POD 1 and discharged home later that day. She had resolution of her left leg pain, with significant improvement in sensation, with only a mild residual numbness of lateral left thigh. Pathology confirmed the intraoperative findings of schwannoma. Postoperative imaging showed gross-total resection and a small, asymptomatic subfascial fluid collection with MR characteristics consistent with seroma. This significantly decreased in size by long-term follow-up.

### 6:52 Series.

We used this technique in a series of 7 patients with intradural extramedullary lesions. Important selection criteria for this technique include posterolateral or ventrolateral location, especially when suspected pathology is a calcified meningioma in the cervicothoracic region. Other ventrally located tumors can be considered, provided the tumor is soft and can be manipulated laterally to facilitate access of the tumor from posterior approach. For lesions at the conus and below, the location of the tumor is generally not a concern due to the ability to manipulate the neural structures (i.e., nerve roots). Additional considerations are size of lesion: our cutoff is about two times the diameter of the tube or one vertebral body segment, especially for dural-based lesions which cannot be manipulated out of the dural defect like the schwannoma seen in the video case. Technical considerations specific for meningioma resection include internal debulking using long, angled ultrasonic aspirator to aid visualization.

All patients were discharged home and had stable or improved functional status postoperatively. At long-term follow-up, we had no instances of clinically relevant CSF leak (i.e., causing wound issues, symptoms, or requiring further intervention), and no other complications or readmissions. Delayed MRI imaging, which was available in a subset of patients, showed a small subfascial fluid collection with MR characteristics consistent with seroma, similar to that seen in the index case and often seen in open cases as well. These collections, again similarly to the index case, remained asymptomatic and significantly decreased in size over time.

In summary, a nonexpandable tubular MIS approach to intradural extramedullary lesions can be used safely and with good outcomes in appropriately selected patients.

## Disclosures

Dr. Lu reported grants from Boston Scientific, Medtronic, and Abbott, outside the submitted work.

## Author Contributions

Primary surgeon: Lu. Assistant surgeon: Harary. Editing and drafting the video and abstract: all authors. Critically revising the work: all authors. Reviewed submitted version of the work: all authors. Approved the final version of the work on behalf of all authors: Lu.

## Supplemental Information

### Patient Informed Consent

The necessary patient informed consent was obtained in this study.
